# Assessment of TSPO in a Rat Experimental Autoimmune Myocarditis Model: A Comparison Study between [^18^F]Fluoromethyl-PBR28 and [^18^F]CB251

**DOI:** 10.3390/ijms19010276

**Published:** 2018-01-17

**Authors:** Ga Ram Kim, Jin Chul Paeng, Jae Ho Jung, Byung Seok Moon, Antonio Lopalco, Nunzio Denora, Byung Chul Lee, Sang Eun Kim

**Affiliations:** 1 Department of Nuclear Medicine, Seoul National University College of Medicine, Seoul National University Bundang Hospital, Seongnam 13620, Korea; kgram@snu.ac.kr (G.R.K.); jaehoboa@paran.com (J.H.J.); bsmoon@snu.ac.kr (B.S.M.); 2 Department of Transdisciplinary Studies, Graduate School of Convergence Science and Technology, Seoul National University, Seoul 16229, Korea; 3 Department of Nuclear Medicine, Seoul National University College of Medicine, Seoul National University Hospital, Seoul 03080, Korea; paengjc@paran.com; 4 Department of Pharmacy–Drug Sciences, University of Bari “Aldo Moro”, Bari 70125, Italy; antonio.lopalco@uniba.it (A.L.); nunzio.denora@uniba.it (N.D.); 5 Center for Nanomolecular Imaging and Innovative Drug Development, Advanced Institutes of Convergence Technology, Suwon 16229, Korea

**Keywords:** myocarditis, noninvasive, positron emission tomography, radiotracer, translocator protein

## Abstract

Overexpression of the 18-kDa translocator protein (TSPO) is closely linked to inflammatory responses in the heart, including myocarditis, which can lead to myocardial necrosis. In vivo assessment of inflammatory responses has enabled the precise diagnosis of myocarditis to improve clinical outcomes. Here, we evaluated TSPO overexpression in a rat model of experimental autoimmune myocarditis (EAM) compared to healthy rats using two TSPO radiotracers, [^18^F]fluoromethyl-PBR28 ([^18^F]**1**) and [^18^F]CB251 ([^18^F]**2**). All radiolabeling methods were successfully applied to an automated module for the reproducible preparation of TSPO radiotracers. Both radiotracers were directly compared in an EAM rat model, as well as in healthy rats to determine whether either radiotracer provides a more promising assessment of in vivo TSPO overexpression. [^18^F]**2** provided more specific TSPO-uptake in the heart of the EAM rats (1.32-fold that of the heart-to-lung uptake ratio versus healthy controls), while [^18^F]**1** did not show a significant difference between the two groups. Histopathological characterization revealed that a prominent positron emission tomography (PET) signal of [^18^F]**2** in the EAM rats corresponded to the presence of a higher density of TSPO compared to the healthy controls. These results suggest that the imidazole[1,2-a]pyridine-based radiotracer [^18^F]**2** is a sensitive tool for noninvasively diagnosing myocarditis related to inflammation of the heart muscle by assessing abnormal TSPO expression.

## 1. Introduction

The 18-kDa translocator protein (TSPO), which is located mainly in the outer membrane of mitochondria, is a well-established biomarker of brain injury and cancer because it is excessively expressed in such lesions [1,2,3]. TSPO regulates cholesterol transport, cell proliferation, apoptosis, and inflammation [4,5]. Particularly, TSPO is important in detecting inflammation in terms of macrophage infiltration. Studies on TSPO are limited to specific diseases such as glioblastoma, neurodegeneration, and prostate and breast cancer [6,7,8]. However, many studies on myocardial inflammation, particularly the role of TSPO in myocarditis, have recently been conducted as TSPO was found to be overexpressed in myocardial inflammatory foci [9,10]. Myocarditis has a relatively low incidence but diagnosis of the disease is difficult at an early stage. The main reason is the similar symptoms to other heart diseases, such as chest pain, myocardial dysfunction, and dyspnea. Thus, myocarditis can be easily confusing with other heart diseases such as myocardial infarction [11,12]. This suggests that myocarditis has clinical variations relying on its symptoms. Furthermore, myocarditis is rapidly pervasive and can be fatal in children and seniors with weakened immune systems compared to the overall population [13,14]. Thus, myocarditis should be diagnosed at an early stage. Cardiac magnetic resonance imaging and echocardiography are conventional imaging methods for detecting myocarditis but show limited sensitivity and specificity [15,16,17]. In contrast, nuclear imaging can be applied to acquire, with high sensitivity, functional information on heart diseases such as myocardial infarction, myocarditis, and cardiomyopathy [18,19]. Using an appropriate radiotracer for targeting inflammatory lesions, the imaging efficiency of myocarditis detection can be maximized. Hence, to enable early diagnosis, we evaluated the feasibility of TSPO-specific radiotracers that can provide functional positron emission tomography (PET) images of the inflammatory response in damaged heart muscle.

Recently, [^125^I]IodoDPA-713, one TSPO radiotracer, has been evaluated for the ability to detect myocardial inflammation in a mouse model of coxsackievirus B3 (CVB3) myocarditis [20,21]. However, direct and effective diagnosis of myocarditis by using [^125^I]IodoDPA-713 has still some limitations (e.g., low heart uptake, high lung uptake, and single photon emission computed tomography). Therefore, we turned our eyes to different structural classes of TSPO radiotracers (e.g., aryloxyanilides such as [^11^C]PBR28, [^18^F]fluromethylPBR28, and [^18^F]FEPPA; imidazopyridine acetamide such as [^18^F]CB251) to noninvasively visualize myocardial inflammation at an early stage.

**Figure 1:**
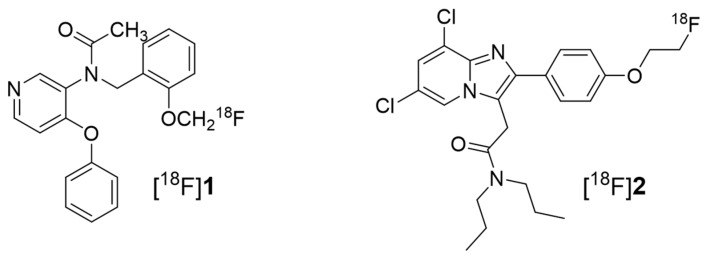
Structure of the 18-kDa translocator protein (TSPO) radiotracers [^18^F]fluoromethyl-PBR28 for [^18^F]**1** and [^18^F]CB251 for [^18^F]**2**.

Here, we describe the in vivo evaluation of two classes of TSPO-selective PET radiotracers (Figure 1) for detecting myocardial inflammatory foci by assessing TSPO expression in a rat model of experimental autoimmune myocarditis (EAM). [^18^F]fluoromethyl-PBR28 ([^18^F]**1**, Ki = 1.85 nM) is an aryloxyanalide analog and displays the inflammatory lesion with an excellent target-to-background ratio at an early imaging time compared to [^11^C]PBR28 [22]. Another TSPO radiotracer, [^18^F]CB251 ([^18^F]**2**, K_i_ = 0.27 nM), is a slightly modified compound from alpidem belonging to the imidazopyridine acetamide class [23]. It was evaluated to detect neuroinflammation as well as TSPO-rich cancer in animal models as a favorable PET radiotracer and has shown specific and reasonable uptake in abnormal TSPO expression lesions. To extend our knowledge, we utilized two different classes of TSPO radiotracers ([^18^F]**1** and **2**) to noninvasively visualize myocardial inflammation at an early stage. In the present study, we propose the application of a TSPO-selective radiotracer and evaluate its suitability for accurate diagnosis of myocarditis.

## 2. Results

### 2.1. Synthesis of the TSPO Radiotracers

The automated production of two radiotracers was performed using the TRACERlab FX N Pro (for [^18^F]**1**) and TRACERlab FX_FN_ (for [^18^F]**2**) module, respectively, with slight modifications based on the reported literature [22,23]. Unlike the reported methods, [^18^F]**1** was prepared in two steps from dibromomethane by nucleophilic aliphatic substitution, which involved the preparation of the radioactive intermediate CH_2_Br[^18^F]F followed by *O*-alkylation of desmethyl-PBR28 [24]. The synthesis of both radiotracers was established as convenient and reliable methods for the biological evaluation of healthy controls and EAM, respectively, as summarized in Table 1.

### 2.2. PET Imaging Study

**Figure 2:**
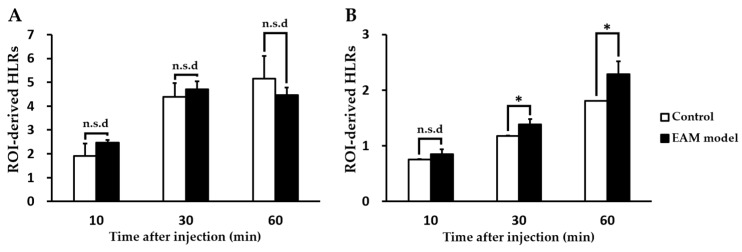
Comparison of incubation time-dependent region of interest (ROI)-derived heart-to-lung ratios of the radiotracers [(**A**) for [^18^F]**1**; (B) for [^18^F]**2**] between healthy and experimental autoimmune myocarditis (EAM) rats (each *n* = 5, respectively). (* *p* < 0.05). n.s.d; no significant difference.

**Figure 3:**
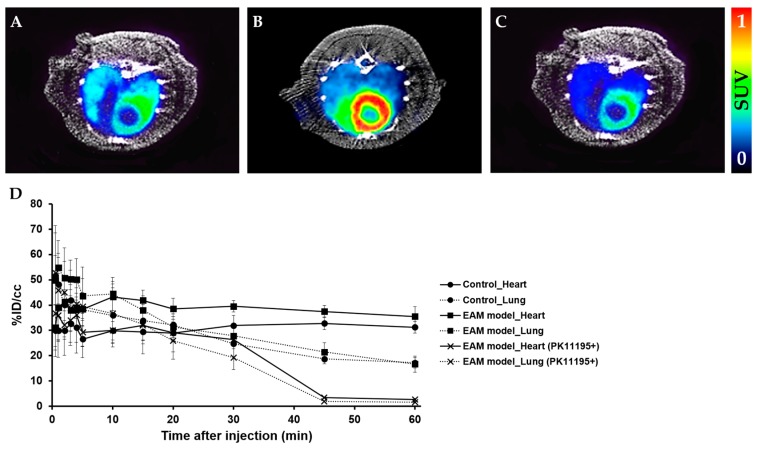
Representative micro positron emission tomography (microPET) axial heart images of [^18^F]**2** in healthy rats and EAM rats at 60 min post-injection. PET images of the heart in the healthy control (**A**), EAM rat (**B**), and EAM rat after PK11195 (10 mg/kg) pre-treatment (**C**) (*n* = 3). Time–activity curves of [^18^F]**2** in the heart and lungs expressed as % ID/cc in three groups (i.e., the healthy rat, EAM rat, and EAM rat (+PK11195)) (**D**).

We evaluated the feasibility of PET imaging by using TSPO radiotracers to noninvasively assess abnormal TSPO expression in the established rat model of myocarditis. In a comparison study, [^18^F]**1** revealed no substantial difference in heart uptake between healthy rat and the EAM model (Figure 2). In contrast, heart-to-lung ratios (HLRs) of [^18^F]**2** were relatively lower than that of [^18^F]**1** but [^18^F]**2** was shown to have significantly increased uptake by the heart; there was also a statistical difference in the HLRs between the two groups. Noninvasive in vivo assessment of TSPO expression in myocarditis showed a 1.32-fold higher heart-to-lung uptake ratio than in the healthy myocardium at 60 min post-injection. In the case of the EAM rats, the influence of cardiac accumulation of [^18^F]**2** is shown in microPET axial heart images in Figure 3A–D. Moreover, the in vivo biodistribution of [^18^F]**2** in heart, expressed as % ID/cc, was significantly increased in the EAM model compared to the healthy control.

To confirm the TSPO-binding specificity of [^18^F]**2**, a blocking experiment was performed. In vivo blocking with excess PK11195 resulted in significantly reduced [^18^F]**2** uptake by both heart and lungs (Figure 3D and Figure S5).

### 2.3. Comparison of TSPO Expression in Myocarditis and Healthy Heart

**Figure 4:**
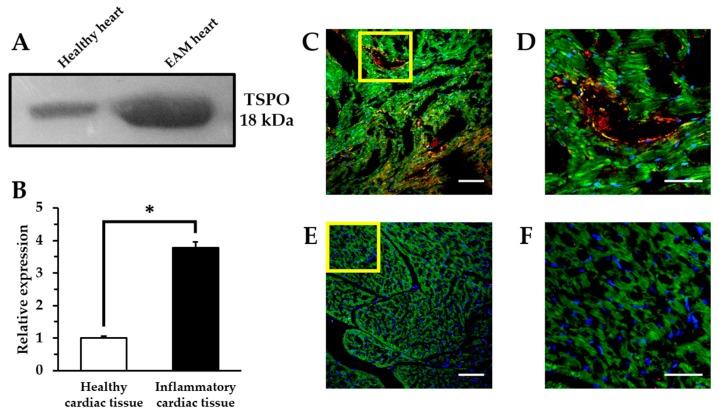
Ex vivo analysis of TSPO expression. Western blotting analysis of heart samples (**A**,**B**). (* *p* < 0.01). Representative immunohistochemical images indicating the expression level of CD68+ macrophages and TSPO in inflammatory myocardial tissue (**C**,**D**) and healthy myocardial tissue (**E**,**F**) by confocal laser scanning microscopy. Highly magnified images of the yellow box in (**C**,**E**), respectively (**D**,**F**). Blue: nucleus; green: mitochondria; red: TSPO; yellow: macrophages. Scale bars: 100 μm (×20) and 50 μm (×60).

We defined the relationship between in vivo [^18^F]**2** accumulation and TSPO levels measured by Western blotting and immunohistochemistry as shown in Figure 4. TSPO expression levels in the heart of the EAM model were approximately 3.7-fold higher than that of the healthy control. Similarly, immunohistochemical results showed that strong fluorescent signals for TSPO were scattered throughout the inflamed myocardium of the EAM model and they were concentrated in the distribution of the macrophages. In contrast, control myocardium showed little fluorescence for TSPO compared to the EAM myocardium. While EAM rats displayed extensive inflammatory foci by numerous macrophage infiltrations, healthy rats showed no inflammatory lesions. Therefore, highly expressed TSPO in the EAM heart tissue can serve as a target for [^18^F]**2** active targeting.

## 3. Discussion

Motivated by our previous results that TSPO radiotracers can sensitively detect TSPO expression in LPS-induced neuroinflammation and tumor models, we further investigated the translation of these findings to other in vivo model related with abnormal TSPO expression. This study revealed that the in vivo potency of a TSPO radiotracer distributed in inflammatory rat heart lesions critically depends on the low nanomolar affinity towards the TSPO. Particularly, the chemical structural differences between the two classes may affect visualization of the results of TSPO expression in inflammatory lesions. To determine whether TSPO radiotracers are selective for assessing TSPO expression in the inflammatory heart, the accuracies of [^18^F]**1** and [^18^F]**2** were checked by comparing the imaging data. HLR analysis was performed since lungs are not affected in the EAM rat in terms of inflammation and fully reasonable as the non-target for TSPO. As expected, HLRs of [^18^F]**2** in the EAM model was 1.32-fold higher than that of the control. In contrast, HLRs of [^18^F]**1** in the EAM model was slightly higher than that in healthy rats until 30 min post-injection but changed reversely at 60 min post-injection. The representative microPET image of [^18^F]**2** shows higher radioactivity in the inflammatory heart of the EAM rat, which visually reflects the 3.7-fold increased TSPO expression in the ex vivo inflammatory heart tissue. Additionally, the time-courses of [^18^F]**2** distribution in lungs (a dotted line) have a similar type of pharmacokinetic profile in both the healthy control and the EAM model but the EAM model showed increased cardiac [^18^F]**2** uptake (a line) compared to the healthy rats. Particularly at an early imaging time, [^18^F]**2** is expected to give the image of an inflammatory lesion with the highest target-to-background ratio due to its rapid approach to a steady state in inflammatory heart lesions. Moreover, the result of the blocking experiment indicates that the uptake of [^18^F]**2** in the EAM model was a specific TSPO-involved immune response against inflammation.

Therefore, these results demonstrate that, compared to [^18^F]**1**, [^18^F]**2** is a desirable radiotracer for assessing the inflammatory response in myocarditis based on its superior binding affinity for TSPO. Our procedure aimed at monitoring the inflammation response related to the abnormal TSPO expression in myocarditis, which causes acute myocardial infarction as well as necrosis. To date, cardiac magnetic resonance imaging and echocardiography have become the gold standard for diagnosis, prognosis, and followup of patients with myocarditis. From our results, much attention must be paid to targeting TSPO for both diagnosis and therapy in the inflammatory heart. However, the molecular imaging strategy to track abnormal TSPO expression in the heart has never been rigorously evaluated by comparison to the current clinical methods. Our data indicate that [^18^F]**2** PET imaging may serve as a promising clinical measure to assess acute TSPO expression in inflammatory heart lesions in more generalized clinical settings. In addition, cardiac TSPO imaging will provide key information in the process of various drug treatments against myocarditis. The effective diagnosis and therapeutic response monitoring would be a valuable approach to precision medicine that can support key steps regarding how myocarditis develops.

In conclusion, we have successfully demonstrated the feasibility of using [^18^F]**2** PET to specifically detect the autoimmune myocarditis at a preclinical stage. We expect in followup studies that early-stage human myocarditis could be diagnosed using [^18^F]**2**.

## 4. Materials and Methods

### 4.1. Synthesis of TSPO Radiotracers

[^18^F]Fluoride was produced at Seoul National University Bundang Hospital using proton bombardment of an ^18^O-enriched water target in the KOTRON-13 cyclotron (Samyoung Unitech Co., Seoul, Korea). The ^18^F-fluoromethyl-PBR28 was synthesized in two steps from dibromomethane by nucleophilic aliphatic substitution, which involved the preparation of the radioactive intermediate ^18^F-CH_2_BrF followed by the *O*-alkylation of desmethyl-PBR28 [24]. The synthesis was performed in the TRACERlab FX N pro (GE Healthcare, Waukesha, WI, USA), which consisted of two reactors. ^18^F was isolated from the enriched water by trapping in a Chromafix-HCO_3_ cartridge previously activated with 2 mL of ethanol and 5 mL of water. After azeotropic distillation of a mixture of acetonitrile (CH_3_CN) and water (1:0.2 mL) dissolved K_2.2.2_/K_2_CO_3_ (15 mg/2.7 mg) by helping additional CH_3_CN (0.3 mL) and a nitrogen stream, a solution of dibromomethane (50 µg) in acetonitrile (1 mL) was subsequently added and the mixture was heated at 120 °C for 5 min. The obtained CH_2_Br[^18^F]F was distilled into a precooled second reactor (−10 °C) containing desmethyl-PBR28 (1 mg) and 5 N NaOH (6 µL) in *N*,*N*-dimethylformamide (DMF, 0.7 mL). The distillation of CH_2_Br[^18^F]F was carried out under 75 mL/min helium flow through in series of four C18 environmental Sep-Pak cartridges. When radioactivity reached a peak, the solution was heated at 100 °C for 5 min. After cooling to approximately 40 °C, the reaction mixture was diluted with 10 mL of water. This solution was loaded into a tC18 Sep-Pak cartridge (Waters, Milford, MA, USA), washed with 10 mL of water, and eluted with 1.5 mL of CH_3_CN. After dilution with 1.5 mL of water, the combined solution was separated by a semi-preparative HPLC system (Xterra RP18, 10 × 250 mm, 45% CH_3_CN/water, flow rate: 3 mL/min) using a UV detector at 254 nm and gamma-ray detector (Bioscan, Poway, CA, USA; Figure S1). The product fraction was collected after approximately 14.5 min and the fraction of [^18^F]**1** collected from the HPLC system was diluted with 20 mL of water. The diluted solution was exchanged to 5–8% ethanol (EtOH)/saline solution by a tC18 Sep-Pak cartridge for further biological evaluation. The final solution was confirmed by an analytical HPLC system (Xterra RP18, 4.6 × 250 mm, 65% CH_3_CN/water, flow rate: 1 mL/min) using a UV detector at 254 nm and gamma-ray detector (Figure S2). The second TSPO radiotracer, [^18^F]**2**, was prepared by nucleophilic aliphatic substitution on a tosylate precursor with fluorine-18 in a single-step radiolabeling procedure as previously described with slight modification [23,25]. The synthesis was performed in the TRACERlab FX_FN_ (GE Healthcare). Briefly, the trapped fluorine-18 in a Chromafix-HCO_3_ cartridge eluted with a mixture of methanol and water (1:0.1 mL) was dissolved with 40% tetrabutylammonium bicarbonate (10 µL). The eluted solution containing fluorine-18 was dried by azeotropic distillation under a nitrogen stream and subsequently mixed with a tosylate precursor (3.0 mg) in *tert*-amylalcohol:DMF (*v*/*v*, 9:1, 1 mL). The reaction mixture was heated to 120 °C for 10 min and cooled to approximately 40 °C. The diluted solution with 10 mL of water was loaded into a C18 plus Sep-Pak cartridge (Waters, USA), washed with 10 mL of water, and eluted with 1.5 mL of CH_3_CN. After dilution with 1.5 mL of water, the combined solution was isolated by a semi-preparative HPLC system (Xterra RP18, 10 × 250 mm, 55% CH_3_CN/water, flow rate: 4 mL/min) using a UV detector (254 nm) and gamma-ray detector (Figure S3). The product fraction was acquired after approximately 31 min and the fraction of [^18^F]**2** was diluted with 40 mL of water. This solution was exchanged to 5–8% EtOH/saline solution by a C18 plus Sep-Pak cartridge for further biological evaluation. The final solution was confirmed by an analytical HPLC system (Xterra RP18, 4.6 × 250 mm, 60% CH_3_CN/water, flow rate: 1 mL/min) using a UV detector at 254 nm and gamma-ray detector (Figure S4).

### 4.2. Experimental Autoimmune Myocarditis (EAM) Rat

Male Lewis rats 7 weeks old weighing 200–250 g were purchased from Orient Biotech (Seoul, Korea) and EAM rats were prepared as previously reported [26,27]. Rats were immunized with purified porcine cardiac myosin (troponin I peptide; Sigma, St. Louis, MO, USA) suspended in an equal volume of complete Freund’s adjuvant (Sigma, USA). This mixture was injected into a footpad once a week.

Before microPET imaging studies, animals were checked with the M-mode echocardiography every week after EAM induction [27]. After 3 weeks of immunization, microPET imaging was performed using EAM rats and healthy controls (each *n* = 5, respectively). All animal experiments in this study were approved by the Seoul National University Bundang Hospital Animal Care and Use Committee (BA1408-158/038-02; approval date: 22 December 2015) and performed in conformity with NIH guidelines (Guide for the care and use of laboratory animals).

### 4.3. Preclinical microPET Imaging Study

**Figure 5:**
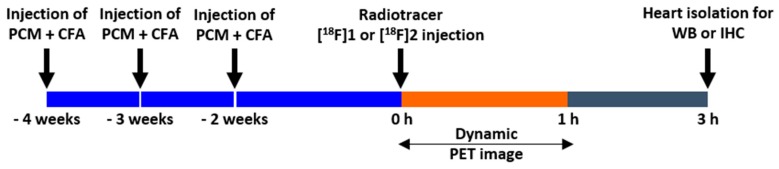
microPET imaging protocol.

Before the scans, the rats were anesthetized with 2% isoflurane in oxygen gas and intravenously injected with 5 to 8% ethanol/saline solution containing 22.2–25.9 MBq (0.5 mL of injection volume) of TSPO radiotracers, [^18^F]**1** or [^18^F]**2** via tail vein, respectively (Figure 5) within 60 min from EOS. The dynamic images were acquired at 60 min post-injection using a NanoScan^®^ PET/CT (Mediso Ltd., Budapest, Hungary). For the blocking study to examine in vivo specificity of uptake by the heart in the EAM rats (*n* = 3), additional PET images of [^18^F]**2** were obtained using the same protocol after treatment of PK11195 (10 mg/kg) 10 min before injection of [^18^F]**2**. At the end of each study, the list-mode data were sorted into dynamic scans consisting of 12 frames. The acquired images were reconstructed by a 3-D Adjoint Monte Carlo method, combined with scatter and random corrections. Reconstructed voxel values in each frame are reported in units of kBq/cc, corrected for radioactive decay to the time of injection. The region of interest (ROI) was used for measuring uptake of lung and heart lesions. We delineated the lung, heart, and myocarditis lesion using PET images of [^18^F]**1** and [^18^F]**2** and the size and axis of the ROIs were adjusted according to the outer contour of each rat’s lung and heart based on its CT images. Regional uptake of radioactivity was decay-corrected to the injection time and was expressed as the standardized uptake value (SUV), which was normalized to the injected radioactivity and animal body weight. ROI was used to semi-quantify the heart-to-lung ratio (HLR) in TSPO imaging with radiotracers [28,29]. The percentage of the injected dose per cubic centimeter of tissue (% ID/cc) was calculated as follows: % ID/cc = ROI activity divided by injected dose multiplied by 100%. The ROIs on the heart and lungs in each group (i.e., the healthy rat, EAM rat, and EAM rat (+PK11195)) after intravenous injection of [^18^F]**2** were manually delineated according to the outer contour of the heart and lung based on its CT images.

### 4.4. Western Blot Analysis

Western blotting (WB) was performed through standard methodology [30]. After PET scans, rats were sacrificed, heart tissues were extracted and protein was isolated from the heart. Protein from each sample was separated by electrophoresis and then electrotransferred to polyvinylidene fluoride membranes. The membranes were immunoblotted with rabbit monoclonal PBR antibody (1:10,000; Novus Biologicals, Littleton, CO, USA) and then with goat-anti-rabbit IgG-HRP (1:5000; Santa Cruz Biotechnology, Dallas, TX, USA). Chemiluminescence (Pierce™ Fast Western Blot Kit, Thermo Fisher Scientific, Waltham, MA, USA) was used for protein detection. The relative band density was analyzed by Fiji ImageJ 2.0.0-rc-64/1.51s software (NIH, Bethesda, MD, USA).

### 4.5. Immunohistochemistry

Heart samples were isolated and fixed with 4% paraformaldehyde and then frozen with OCT compound at −80 °C. For immunohistochemistry (IHC), tissues were sectioned (7 μm thickness). All slices were placed in 0.2% Tween 20 for 10 min, in 3% sodium deoxycholate solution on a shaker for 2–4 h at 37 °C, and in 20–50% normal goat serum in 1% bovine serum albumin-PBS solution for 2 h at 37 °C. Staining with primary antibodies was conducted using CD68 (1:150; AbD Serotec, Hercules, CA, USA) for macrophages and PBR (1:100; Abcam, Cambridge, UK) for TSPO at 4 °C overnight. Finally, the slices were washed and incubated with goat anti-rat IgG antibody (conjugated with Alexa Fluor 488) (1:400; Life Technologies, Carlsbad, CA, USA) for CD68, goat anti-rabbit IgG antibody (Alexa Fluor 647) (1:400; Life Technologies) for PBR, Hoechst dye (1:750; Life Technologies) for nuclear stain, and MitoTracker^®^ Orange CM-2TMRos (Life Technologies) for mitochondria stain, serially. Each stained slide was mounted with gel mount solution (Biomeda Corporation, Foster City, CA, USA). Fluorescence images were acquired with an A1 Rsi confocal laser scanning microscope (Nikon, Tokyo, Japan).

### 4.6. Statistical Analysis

Comparison uptake of radiotracers and quantification of TSPO expression between groups were performed using IBM SPSS Statistics 19. All data were analyzed using an unpaired Students *t*-test. Differences with a *p*-value less than 0.05 were considered as significant.

## Figures and Tables

**Table 1:** Synopsis of the production of two TSPO radiotracers ([^18^F]fluoromethly-PBR28 for [^18^F]**1** and [^18^F]CB251 for [^18^F]**2**).

	Specification	[^18^F]1	[^18^F]2
Used synthetic module	N/A	TRACERlab FX N Pro	TRACERlab FX_FN_
Radiochemical yield (%) ^a^	N/A	9.4 ± 3.7 (*n* = 22)	19.7 ± 2.9 (*n* = 31)
Radiochemical purity	>95%	>99%	>98%
Visual inspection	Clear, colorless, no precipitate	Passes	Passes
Radionuclide purity	511, 1022 KeV	Passes	Passes
Radionuclide identity	105–115 min	Passes	Passes
pH	4.5–8.5	6.0–7.0	6.0–7.0
Residual solvent analysis	acetone <5000 ppm acetonitrile <410 ppm ethanol <100,000 ppm	<45 ppm <48 ppm <72,000 ppm	<35 ppm <50 ppm <72,000 ppm
Molar activity (GBq/µmol) ^b^	>37	146 ± 39	164 ± 52
Product (GBq)/Total volume	N/A	2.88 ± 1.2/10 mL	4.63 ± 0.7/10 mL
Total synthesis time (min)	N/A	95 ± 1	70 ± 1

^a^ Data reported are decay corrected at EOS and represent the mean and standard, ^b^ Molar activities are measured by analytical HPLC at EOS based on integration of the UV area after reinjection of the final solution.

## Supplementary Materials

Supplementary materials can be found at www.mdpi.com/1422-0067/19/1/276/s1.

## Abbreviations

TSPO	18-kDa mitochondrial translocator protein
PBR	peripheral benzodiazepine receptor
PET	positron emission computed tomography
EAM	experimental autoimmune myocarditis
WB	Western blot
IHC	immunohistochemistry
