# Supplementary material for: Assessment of TSPO in a Rat Experimental Autoimmune Myocarditis Model: A Comparison Study between [18F]Fluoromethyl-PBR28 and [18F]CB251
Source: Int J Mol Sci. 2018 Jan 17;19(1):276. doi: 10.3390/ijms19010276 (PMC5796222; doi:10.3390/ijms19010276)
Supplement: Supplementary file 1 [file ijms-19-00276-s001.pdf]

Supplementary Information

Assessment of TSPO in a Rat Experimental Autoimmune Myocarditis Model: A Comparison Study between [^{18}F]Fluoromethyl-PBR28 and [^{18}F]CB251

Ga Ram Kim, Jin Chul Paeng, Jae Ho Jung, Byung Seok Moon, Antonio Lopalco, Nunzio Denora,

Byung Chul Lee* and Sang Eun Kim*

I. Representative HPLC chromatogram for [^{18}F]Fluoromethyl-PBR28

II. Representative HPLC chromatogram for [^{18}F]CB251

III. *In vitro* binding assay

IV. PET images based time-activity curves analysis

V. Reference

I. Representative HPLC chromatogram for [^{18}F]Fluoromethyl-PBR28

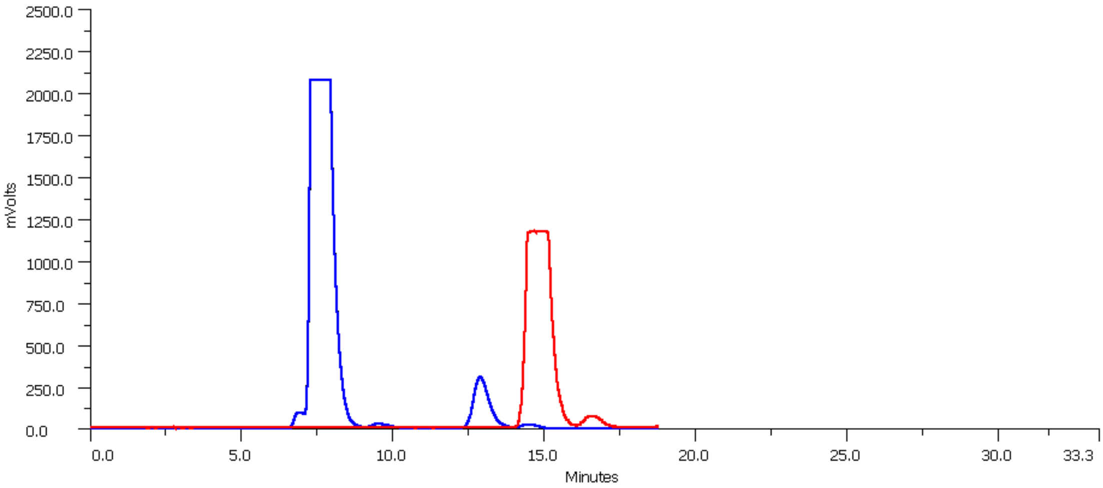

Fig. S1 Preparative-HPLC chromatogram of [^{18}F]Fluoromethyl-PBR28 ([^{18}F]**1**). Column: Xterra RP-18, 10 μm, 10 × 250 mm with a guard cartridge (Phenomenex, 10 × 10 mm); Eluent: 45% CH_3CN/H_2O; Flow rate: 3 mL/min; Blue line: UV-254 nm; Red line: gamma-ray.

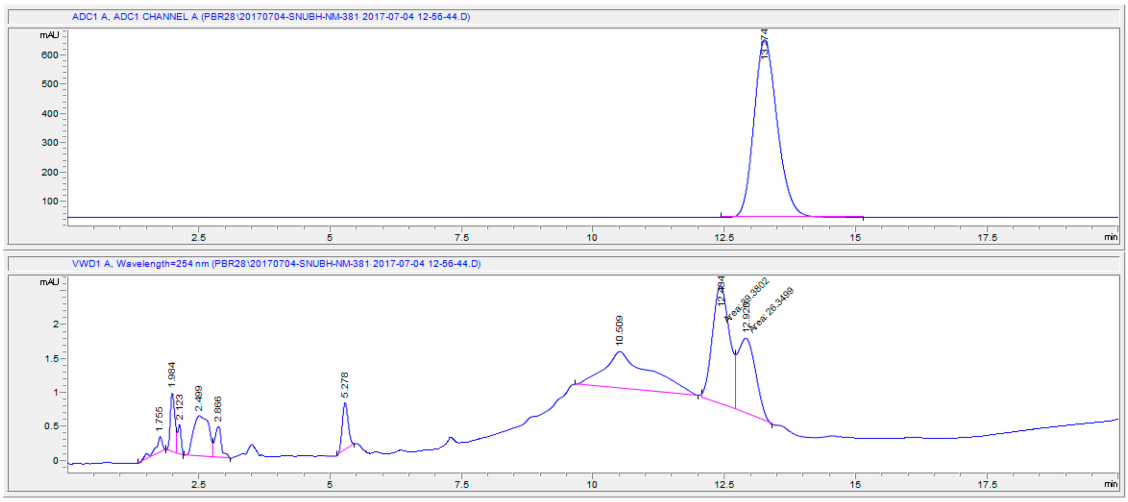

Fig. S2 Analytical HPLC chromatogram of pure [^{18}F]Fluoromethyl-PBR28 ([^{18}F]**1**). Column: Xterra RP-18, 5 μm, 4.6 × 250 mm; Eluent: 65% CH_3CN/H_2O; Flow rate: 1 mL/min; Upper: gamma-ray; Bottom: UV-254 nm.

II. Representative HPLC chromatogram for [^{18}F]CB251

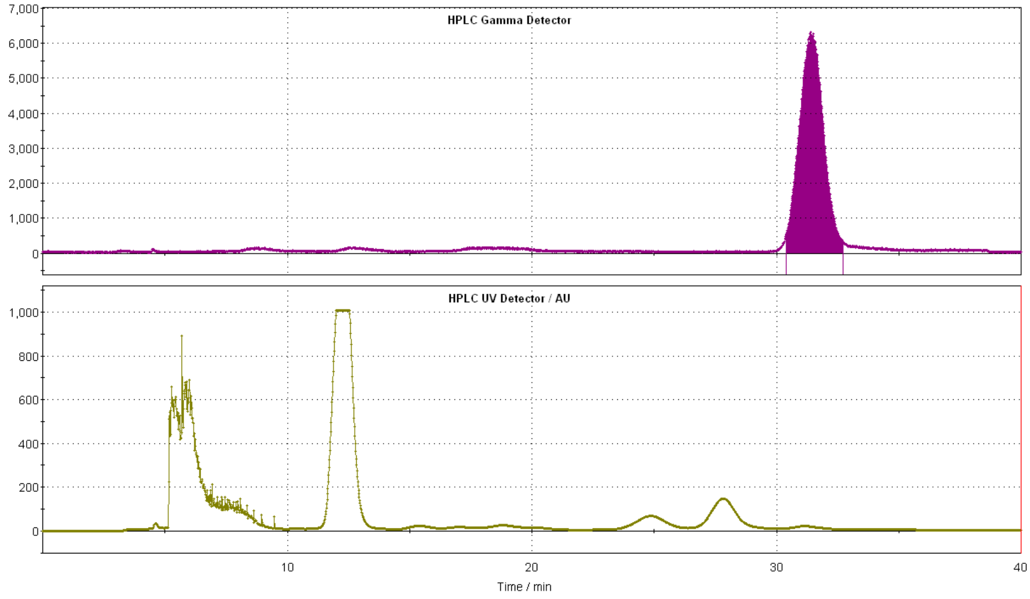

Fig. S3 Preparative-HPLC chromatogram of [^{18}F]CB251 ([^{18}F]**2**). Column: Xterra RP-18, 10 µm, 10 × 250 mm with a guard cartridge (Phenomenex, 10 × 10 mm); Eluent: 55% CH_3CN/H_2O; Flow rate: 4 mL/min; Bottom: UV-254 nm; Upper: gamma-ray.

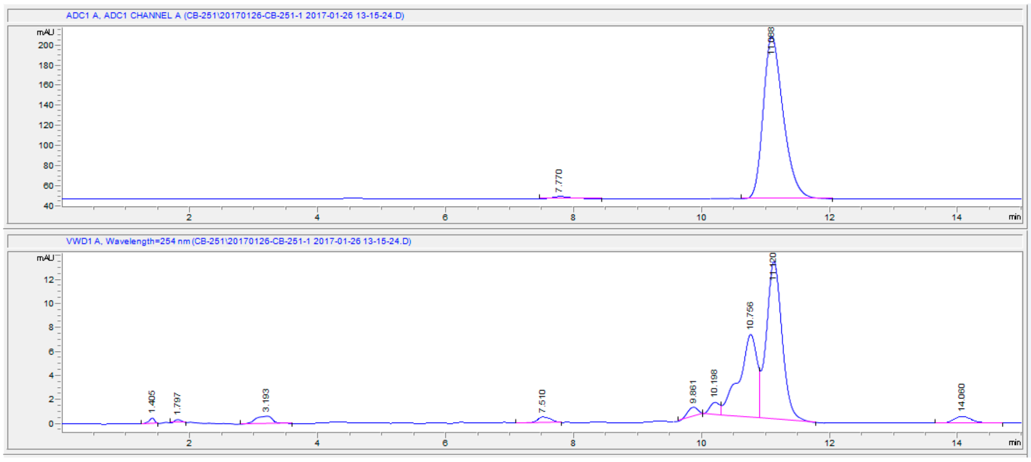

Fig. S4 Analytical HPLC chromatogram of pure [^{18}F]CB251 ([^{18}F]**2**). Column: Xterra RP-18, 5 µm, 4.6 × 250 mm; Eluent: 60% CH_3CN/H_2O; Flow rate: 1 mL/min; Upper: gamma-ray; Bottom: UV-254 nm.

III. *In vitro* TSPO binding assay

Method

Binding affinity to the TSPO was assessed using *in vitro* receptor binding assay. This analysis was conducted as previously described by Denora et al [1].

Result

The binding affinities of fluoromethyl-PBR28 and CB251 for TSPO and CBR, expressed as inhibition constants (K_i), were compared with those of unlabeled PK11195. Fluoromethyl-PBR28 was characterized by a binding affinity for TSPO (K_i = 1.85 nM) while CB251 showed a subnanomolar binding affinity for TSPO (K_i = 0.27 nM).

IV. PET images based time-activity curves analysis

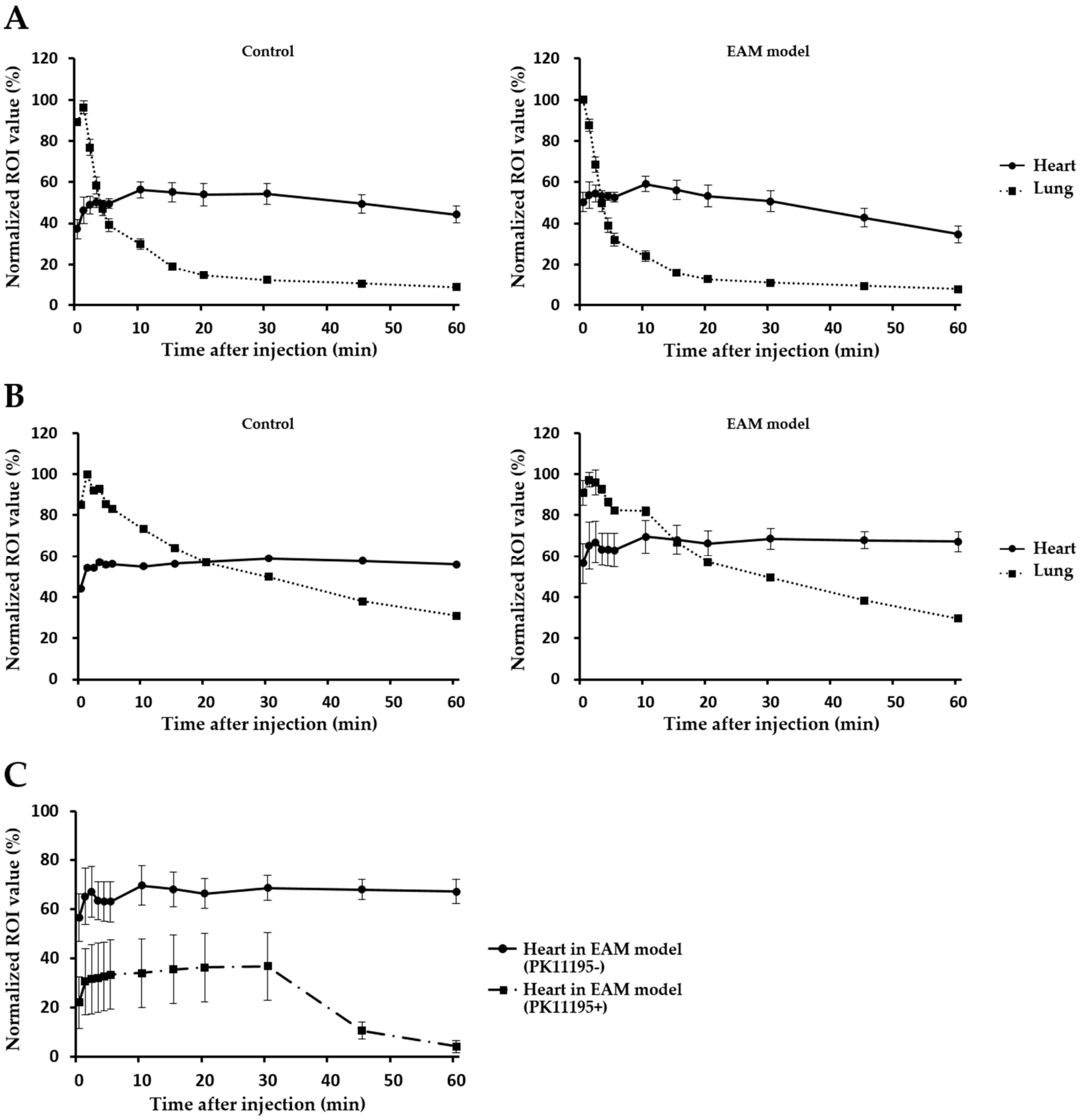

Fig. S5 MicroPET time-activity curves for [^{18}F]fluoromethyl-PBR28 ([^{18}F]**1**) and [^{18}F]CB251 ([^{18}F]**2**) in control (n = 5) and EAM model (n = 5) during 60 min after injection. Time-activity curves with [^{18}F]**1** (A), [^{18}F]**2** (B), and PK11195 (10 mg/kg, n = 3) treatment before injection of [^{18}F]**2** (C). The time–activity curves for the heart and lung are plotted 1 min after injection of [^{18}F]**1** or [^{18}F]**2**. The uptake values in ROI were normalized for the peak uptake value of lung.

V. Reference

1. Denora, N.; Laquintana, V.; Trapani, A.; Lopedota, A.; Latrofa, A.; Gallo, J.M.; Trapani, G. Translocator protein (TSPO) ligand-Ara-C (cytarabine) conjugates as a strategy to deliver antineoplastic drugs and to enhance drug clinical potential. *Mol Pharm* **2010**, *7*, 2255-2269.
